# *Angiostrongylus chabaudi* Biocca, 1957: a new parasite for domestic cats?

**DOI:** 10.1186/s13071-014-0588-1

**Published:** 2014-12-17

**Authors:** Antonio Varcasia, Claudia Tamponi, Emanuele Brianti, Piera Angela Cabras, Roberta Boi, Anna Paola Pipia, Alessio Giannelli, Domenico Otranto, Antonio Scala

**Affiliations:** Laboratorio di Parassitologia, Ospedale Didattico Veterinario, Dipartimento di Medicina Veterinaria, Università degli Studi di Sassari, 07100 Sassari, Italy; Dipartimento di Scienze Veterinarie, Università degli Studi di Messina, 98168 Messina, Italy; Istituto Zooprofilattico Sperimentale della Sardegna, 08048 Tortolì, Italy; Dipartimento di Medicina Veterinaria, Università degli Studi di Bari, 70010 Valenzano, Bari Italy

**Keywords:** *Angiostrongylus chabaudi*, Metastrongyloidea, Angiostrongylidae, Cat, Cardio-pulmonary nematodes

## Abstract

**Background:**

Natural infection with a species of *Angiostrongylus* has been reported only once in wildcats from central Italy by Biocca in 1957. The causative species of this infection was identified as *Angiostrongylus chabaudi*. Following this report, this parasite had never been found in either wild or domestic cats.

**Findings:**

The lungs and the pulmonary arteries of an adult female cat (*Felis silvestris catus*), road-killed in Sardinia, Italy, were macroscopically examined and dissected under a light microscope for the presence of parasites. A slender nematode was detected and its morphometrical features were consistent with those of *A. chabaudi*. Morphological data were supplemented by sequencing of the partial cytochrome oxidase *c* subunit 1 (*cox*1) gene, as well as the internal transcribed spacer 2 (ITS2) of the rDNA. Nucleotide sequences displayed 99% homology with the ITS2 sequence [GenBank KM216825.1] of a specimen of *Angiostrongylus* sp. recovered recently from the pulmonary artery of a wildcat in Germany and 91% with *cox*1 sequence [GenBank GU138118.1] of *Angiostrongylus vasorum*.

**Conclusion:**

The results of the present study indicate, for the first time, that *A. chabaudi* may also infect domestic cats, and thus should be considered in the diagnosis of metastrongyloid species infecting their cardio-pulmonary system.

## Findings

Nematodes affecting the cardio-pulmonary system of pets have recently attracted the interest of researchers due to their increasing distribution in several European countries [1]. This is the case of *Aelurostrongylus abstrusus* and *Eucoleus aerophilus* (syn. *Capillaria aerophila*) in cats and of *Angiostrongylus vasorum* in dogs [2]. Recent reports of infections caused by other members of the superfamily Metastrongyloidea in domestic cats (e.g., *Oslerus rostratus*, *Troglostrongylus brevior* and *Troglostrongylus subcrenatus*) have further stimulated the interest of the scientific community on these little known species [3-7]. In addition, cats may act as permissive hosts of *A. vasorum*, although the first-stage larvae of this nematode are not shed in the faeces of experimentally infected animals [8,9]. A natural infection with a species of *Angiostrongylus* has been reported only once in wildcats from central Italy by Biocca in 1957, who described and named the causative species as *Angiostrongylus chabaudi* [10]. Following this report, the species has never been found in either wild or domestic cats. Fifty-seven years after its description, we report the presence of *A. chabaudi* in a domestic cat from Sardinia together with the first molecular characterization of this little known angiostrongylid infecting cats.

The material originated from an adult female cat, road-killed in the municipality of Villacidro, province of Cagliari, Sardinia, Italy (39°27’33”N, 8°44’02E”). Upon inspection of the pulmonary arteries, a slender nematode was retrieved. This parasite was washed in saline solution and subsequently mounted on a slide with glycerol, for microscopical observation. Light microscopy images and measurements were taken using a digital image processing system (Olympus BX41; Soft Imaging solution GMBH LG20, Munster, Germany). The parasite was identified to the species level using morphological keys [10,11]. A faecal sample was also collected from the rectum of the cat and processed by Baermann method for the detection of first-stage larvae of broncho-pulmonary nematodes [7]. The genetic identity of the cat (i.e., *Felis silvestris silvestris*, *Felis silvestris lybica*, *Felis silvestris catus* or hybrid) was assessed molecularly on a muscle sample as described elsewhere [12].

Following parasite identification, DNA was extracted from the mid-body of the nematode using a commercial kit (High Pure PCR Template Preparation kit, Roche Diagnostics, Mannheim, Germany). Partial mithocondrial cytochrome *c* oxidase subunit 1 (*cox*1) gene and the internal transcribed spacer 2 (ITS2) of the rRNA gene were amplified, as previously described [13,14]. PCR amplicons were purified using an Exo SAP-IT kit (Amersham Biosciences) and sequenced through an external service (MWG Eurofins), using the same primers as for the PCR. Nucleotide sequences were compared with those available in GenBank® using the basic local alignment search tool (BLAST) analysis (National Centre for Biotechnology Information, ncbi.nlm.nih.gov). In order to investigate the relationships among metastrongyloids affecting wild and domestic carnivores, sequences of *cox*1 and ITS2 were analysed with those available in GenBank™. The evolutionary history was inferred for ITS2, using the maximum parsimony method using the Subtree-Pruning-Regrafting (SPR) algorithm with the software MEGA 6 [15]. The bootstrap consensus trees inferred from over 8,000 replicates were taken to represent the evolutionary history of the taxa analysed. The ITS2 sequence of *Nematodirus battus* was used as an outgroup.

**Figure 1:**
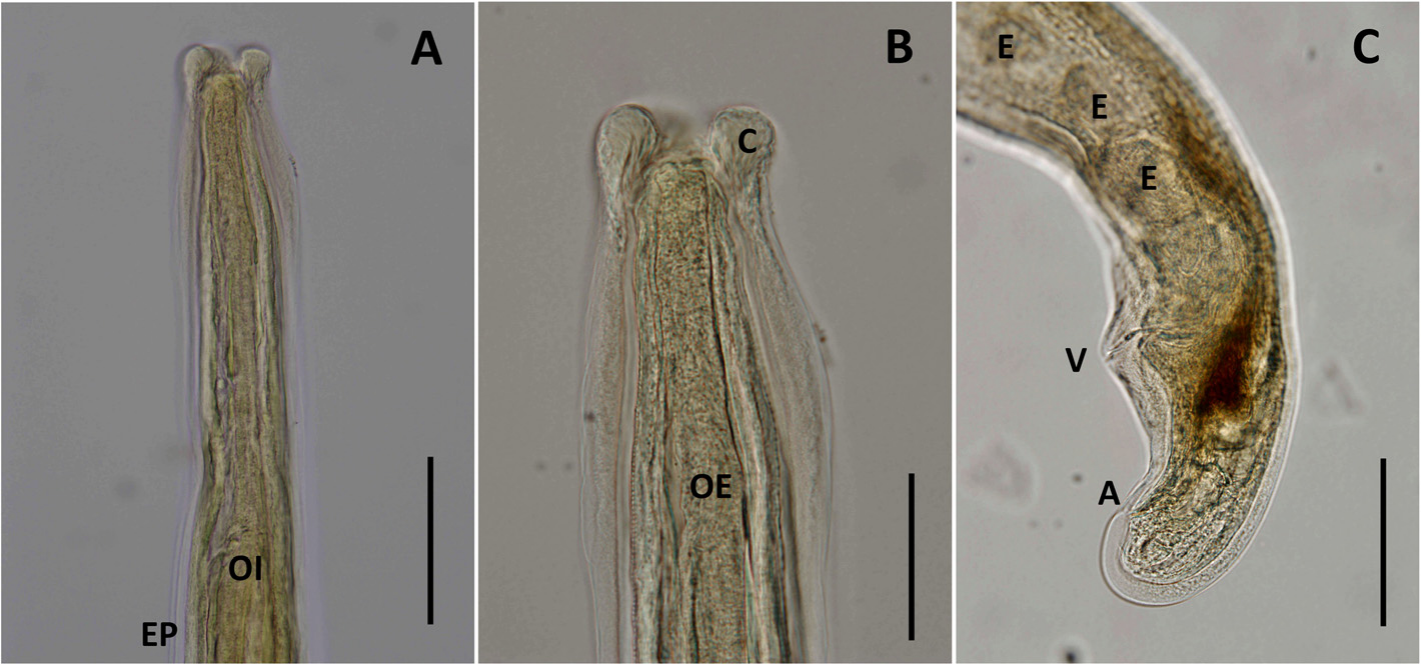
***Angiostrongylus chabaudi.*** Light microscopy photomicrographs of female, lateral view. **A**. Cephalic region in lateral view; note the oesophageal-intestinal junction (OI) and level of the excretory pore (EP). **B**. Apical end, note dilatation of the cuticle resembling cephalic alae (C). **C**. Caudal end; note presence of eggs (E), vulvar aperture (V) and anus (A). Scale bars: **A** = 50 μm; **B**–**C** = 100 μm.

**Figure 2:**
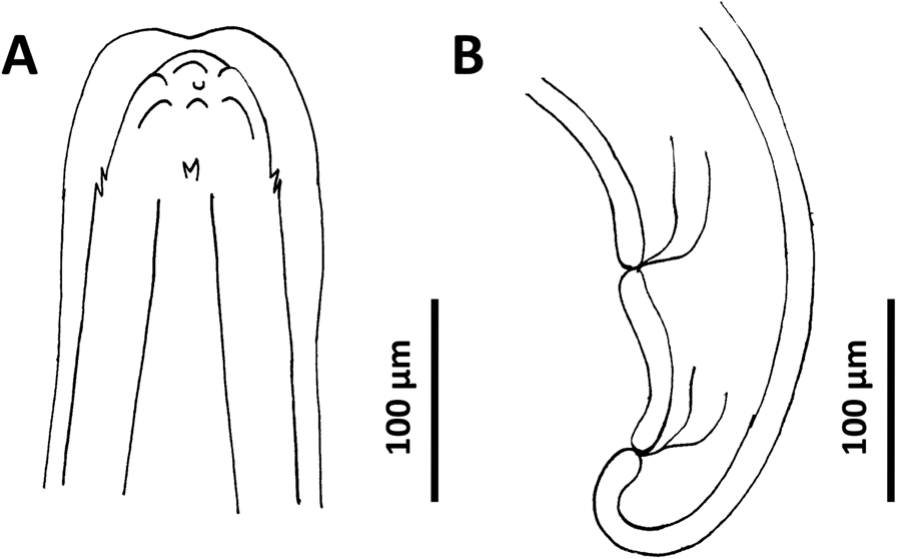
***Angiostrongylus chabaudi*** **, female.** Schematic drawings. **A**. Anterior end, lateral view. **B**. Caudal end, lateral view.

The parasite specimen was a female with a slender and elongated body (16.9 mm long and 195.8 μm wide). The anterior extremity was attenuated, whilst the posterior was rounded and curved forward (Figure 1). The cuticle was longitudinally striated and slightly dilated in the anterior end (Figure 1B). The buccal aperture, small and circular, was situated in the terminal position, surrounded by six perityls (Figure 2A). Two pairs of sensorial papillae (two ventral and two dorsal), each represented by two symmetrical conical protuberances, were noted posteriorly to the perityls (Figure 2A). The oesophagus was 276.6 μm long and clavated. The excretory pore opened just behind the oesophageal-intestinal junction, at 361.5 μm from the apical end. The vulva was sub-terminal and the anus was posterior to the vulvar aperture at 160.6 μm and at 43.4 μm, respectively, from the caudal end (Figures 1C and 2B). Eggs (mean length 49.6 ± 4.2 μm; mean width 33.4 ± 0.9 μm) were seen inside the terminal tract of the uterus and in the ovijector (Figure 1C). The morphometrical features of the above parasite were consistent with those of *A. chabaudi* (Table 1) reported in the original description [10]. At the Baermann examination, only first-stage larvae of *Troglostrongylus brevior* were found and molecularly confirmed (data not shown). According to the molecular identification of the genotype, the cat was identified as pure domestic cat (*F. silvestris catus*).

**Table 1:** **Measurements for the known species of** ***Angiostrongylus*** **reported in carnivores**

**Species**	**Present specimen**	***Angiostrongylus chabaudi*** [10]	***Angiostrongylus*** **sp.** [16]	***Angiostrongylus*** **sp.** [16]	***Angiostrongylus vasorum*** [18]	***Angiostrongylus daskalovi*** [18]	***Angiostrongylus gubernaculatus*** [18]
**Host(s)**	**Domestic cat**	**Wildcat**	**Fox**	**Badger**	**Dog, Coyote, Fox, Jackal, Wolf**	**Badger, Pine marten, Beech marten**	**American badger, Striped skunk**
*Males*							
Length (mm)	-	14.6 - 16.3	13.90 ± 4.75 (n = 11)	19.36 ± 7.69 (n = 6)	14.0 - 15.5	13.36 - 21.31	18 - 19.5
Width (maximum)	-	185 - 225	221.40 ± 12.13 (n = 11)	243.20 ± 19.63 (n = 6)	170 - 235	254 - 306	300 - 335
Oesophagus length	-	300 - 345	255.33 ± 13.60 (n = 8)	333.30 ± 18.24 (n = 6)	220 - 275	336 - 366	300 - 325
Distance from excretory pore to cephalic end	-	335 - 405	356.50 ± 34.68 (n = 4)	409.5 ± 25.50 (n = 2)	310 - 350	386 - 463	-
Spicules length	-	510 - 555	411.06 ± 17.71 (n = 10)	345.57 ± 23.95 (n = 8)	400 - 500	336 - 409	520 - 560
*Females*							
Length (mm)	16.9	19.1 - 24.1	18.97 ± 10.21 (n = 22)	24.76 ± 13.66 (n = 16)	15 - 20.5	14.39 - 31.12	22 - 24
Width (maximum)	192.2	245 - 298	304.14 ± 14.84 (n = 18)	344.52 ± 17.95 (n = 17)	220 - 306	340 - 511	350
Oesophagus length	276.6	345 - 380	279.88 ± 20.27 (n = 14)	368.45 ± 17.00 (n = 16)	240 - 280	356 - 556	335 - 350
Distance from excretory pore to cephalic end	361.5	395 - 470	391.86 ± 21.44 (n = 7)	447.40 ± 101.72 (n = 5)	350 - 370	379 - 636	-
Distance from vulva to anus	117.2	-	233.63 ± 32.41 (n = 17)	295.15 ± 46.25 (n = 16)	150 - 220	-	-
Distance from anus to caudal end	43.4	62 - 75	69.70 ± 2.87 (n = 17)	78.82 ± 3.45 (n = 16)	67 - 100	76 - 115	75 - 95
Distance from vulva to caudal end	160.6	170 - 210	275.66 ± 30.75 (n = 18)	366.25 ± 44.17 (n = 16)	220 - 315	269 - 412	-

All measurements are given in micrometers (μm) unless otherwise specified.

The BLASTn of *cox*1 sequences herein obtained displayed a nucleotide homology of 91% with *A. vasorum* [GenBank GU138118.1], 89% with *Angiostrongylus cantonensis* [GenBank GU138111.1] 88% with *A. abstrusus* [GenBank KF316481.1] and of 87% with *Angiostrongylus costaricensis* [GenBank GU138117.1]. Interestingly, the ITS2 sequence displayed 99% nucleotide identity with ITS2 of *Angiostrongylus* sp*.* [GenBank KM216825.1], recovered recently from the pulmonary artery of a wildcat in Germany and identity of 87% with *A. vasorum* [GenBank GU045375.1]. The ITS2 sequence of *A. chabaudi* displayed lower levels of homology of 79%, 85% and 79%, respectively, with *A. dujardini* [GenBank GQ181113.1], *A. costaricensis* [GenBank LK942974.1] and *A. cantonensis* [GenBank LK949842.1].

**Figure 3:**
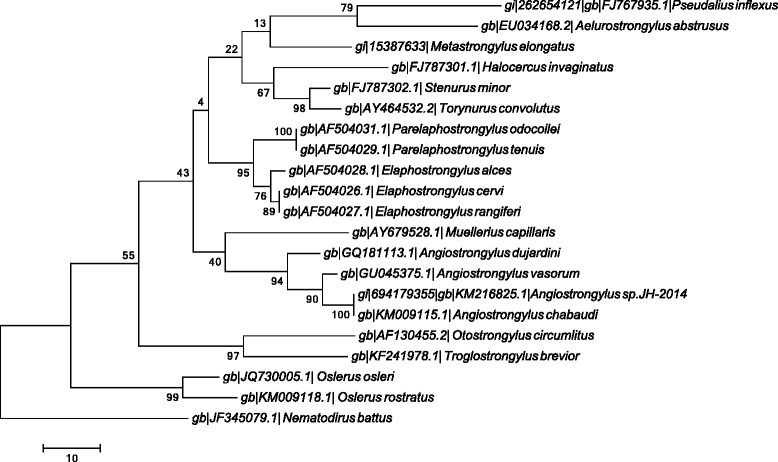
**Phylogenetic tree based on ITS2 sequences for different species of metastrongyloid nematodes retrieved from GenBank.** The evolutionary history was inferred using the Maximum Parsimony method. The tree was obtained using the Subtree-Pruning-Regrafting (SPR) algorithm.

Pairwise distances, calculated using the common region of *cox*1 (454 bp) varied from 0.08 with *A. vasorum* [GenBank EU493161.1] to 0.13 with both *A. cantonensis* [GenBank GU138111.1] and *A. abstrusus* [GenBank KF316481.1] and of 0.17 with *A. costaricensis* [GenBank GU138116.1], whereas those based on ITS2 were identical with a sequence for *Angiostrongylus* sp. [GenBank KM216825.1] and diverged by 0.08 from that for *A. vasorum* [GenBank GU045375.1]. Analysis of the *cox*1 sequences clustered *A. chabaudi* in the clade including *A. vasorum* but this was not optimal due to the small number of sequences available (data not shown). Conversely, ITS2 phylogeny clearly indicated that *A. chabaudi* clustered within the clade of all other *Angiostrongylus* species available in public databases (Figure 3).

Fifty-seven years after its original description [10], *A. chabaudi* is reported here for the first time in a domestic cat from Sardinia. In addition, the present study represents the first report, ever, of an angiostrongylid of the genus *Angiostrongylus* naturally infecting domestic cat. The recovery of this nematode is of importance as it suggests that cats may also be infected by metastrongyloids localizing in the cardio-vascular system. Morphological features and size of the parasite found in the present survey are similar to those reported in the original description of *A. chabaudi* [10] (Table 1). Although eggs were observed in the uterus of the specimen herein studied, the absence of specific first-stage larvae at the Baermann test indicates that the parasite was an immature or unfertilized female, thereby explaining its smaller size compared to that previously recorded [10]. The morphometry of the parasite here described differs from that of *A. vasorum* in dogs and wildlife hosts (Table 1) [16], in that four sensorial papillae are present in the anterior region posteriorly to perityls, and the distance between the vulvar aperture and the caudal extremity (i.e., 160.6 μm) is significantly shorter than in other species of *Angiostrongylus* but similar to that of *A. chabaudi* (170-210 μm) (Table 1). These morphological differences were also clearly supported by the nucleotide homology with sequences from other species (i.e., up to 91% for *cox*1 sequences with that of *A. vasorum*). The phylogenetic analyses, based on ITS2, supported their morphological identification, in that sequences derived from this nematode were clustered within those belonging to the genus *Angiostrongylus*.

In the original description, *A. chabaudi* was considered typical for wildcats, having been found in 85% of a sample from central Italy, but not in stray cats and dogs or in other wildlife species (i.e., *Meles meles* and *Vulpes vulpes*) from the same area [10].

Here *A. chabaudi* is reported for the first time in a domestic cat. No data are available on the presence of this nematode in wildcats from Sardinia, which belong to another subspecies (*F. silvestris lybica*) than that found in continental Italy [17]. Therefore, at least for Sardinia, it is not yet possible to infer that wildcats may be regarded as wild reservoirs of *A. chabaudi*. On the other hand, according to Biocca’s study [10], wildcats may be highly infected by *A. chabaudi*. In addition, the ITS2 sequence of *A. chabaudi* herein characterized displayed a very high nucleotide homology (99%) with that of *Angiostrongylus* sp. collected from a wildcat in Germany*.* Also, both nematodes above were collected from the pulmonary artery, which is the same site of collection of *A. chabaudi* as described by Biocca [10].

The data presented here suggest that a careful inspection of the vascular system of lungs should be undertaken during necropsies of domestic and wild cats. Also, first-stage larvae of *A. chabaudi* have not been described so far [10] and thus their morphological delineation from those of other metastrongyloids affecting felids warrants investigation. Additional studies should elucidate the life history of *A. chabaudi*, determine its distribution and impact of this little known parasite on the health of wild and domestic cats.
